# Moral controversies and academic public health: Notes on navigating and surviving academic freedom challenges

**DOI:** 10.1016/j.gloepi.2023.100119

**Published:** 2023-08-15

**Authors:** Tyler J. VanderWeele

**Affiliations:** Harvard T. H. Chan School of Public Health, 677 Huntington Avenue, Boston, MA 02115, United States

## Abstract

Schools of public health often serve both as public health advocacy organizations and as academic units within a university. These two roles, however, can sometimes come into conflict. I experienced this conflict directly at the Harvard T. H. Chan School of Public Health in holding and expressing unpopular minority viewpoints on certain moral controversies. In this essay I describe my experiences and their relation to questions of academic freedom, population health promotion, and efforts at working together across differing moral systems.

## 1 Introduction

The Harvard T. H. Chan School of Public Health (HSPH) has been my academic home for eighteen years, first for four years as a doctoral student, and then later these past fourteen years as a faculty member. Over those years, the School has provided a stimulating and supportive environment. However, during the past months, various events have altered my experience and understanding of the School. In March of 2023, a series of Twitter posts were published by public health academics, principally concerning an *amicus curiae* brief I had signed in 2015 [1] in the Obergefell vs. Hodges case in the Supreme Court. The Brief argued that (i) there were two competing views of marriage at play, one more grounded in procreation and providing a stable family environment for children, and another more focused on the bond and personal fulfillment of partners; that (ii) the Constitution itself did not specify a view of marriage, and thus that (iii) it would be better if the matter were taken up by the states and their people rather than by the courts. My signing of the brief was linked in the Twitter posts to a commentary that I had published in *JAMA Psychiatry* on abortion and mental health [2]. That commentary had argued that the abortion and mental health literature had been weaponized by both sides of the abortion policy debate; that the moral contours of the policy debate lay elsewhere concerning the moral status of a fetus on the one hand versus autonomy, control, privacy and the rights of women on the other; and that the abortion and mental health literature should thus be more oriented towards providing for the mental health needs of women regardless of their views. The Twitter posts led to turmoil at HSPH including calls for my tenure to be revoked and for me to be fired, along with public condemnations of my views by prominent academic administrators.

In this essay, I would like to describe the course of events; consider whether the positions that were the source of controversy should be admissible within academic public health; and take up the issues of academic freedom, viewpoint diversity, and their relation to broader society and public health efforts. While the events described here are of course very specific, they bring up issues that are more general [[3], [4], [5], [6]]. They give rise to questions concerning the extent to which a research university is able to facilitate a free exchange of potentially opposing ideas within the context of intellectual diversity and civil discourse, and the extent to which university administrators are willing to publicly support the university in this role. This seems especially important within public health, in a context in which there appears to be growing alienation between many academics and billions of others throughout the world who hold differing views on a number of important issues. I will explicitly take up these matters in the second and third sections of this essay, but will first give a personal account of the events, as I experienced them, that gave rise to these reflections and concerns.

## 2 Events at HSPH

The Twitter posts began to be published on March 11th, 2023, and although they centered on the *amicus* brief from 2015, there was also considerable slander towards me, innuendo, and general disparagement. Most of the activity died down within a couple of days, but it reached over 40,000 viewers. The posts were accompanied by e-mails to my colleagues asking if they knew that I had signed the brief, and modifications made to my Wikipedia page, highlighting my signing. Given the extent of the social media posts, it also reached a number of HSPH students. The brief and the *JAMA Psychiatry* commentary were then further linked, as being considered problematic writing, to one of my blog posts in *Psychology Today* about the decline in well-being among youth [7], which had upset some students the prior fall. Two sentences in that blog post raised the question of whether introducing issues of gender identity in the general curriculum as early as kindergarten was conducive to well-being. A number of students at HSPH were very upset by these writings and some seemed to view them as threats to their identity. Within a short timeframe, some students were calling for my tenure to be revoked and that I be fired; or that I be removed from my teaching position of a required quantitative methods course; or that the School take positions on these various issues. Some students indicated that if they had known my views, then they would have refused to attend my quantitative methods class, and, instead, would have organized to protest. On March 17th, in my first conversation with the HSPH administration on these matters, the chair of the Department of Epidemiology indicated that what I had written and signed was within the bounds of academic freedom and that the HSPH Academic Dean had affirmed the same.

During the week of March 20–24, the Population Health Sciences (PHS) PhD Program, in which I teach, hosted a listening session for the students, as did the Department of Epidemiology. At the second of those listening sessions, some students stated that my signing should not be protected by academic freedom. The Dean of Education and Chief Diversity, Inclusion, and Belonging Officer requested a meeting, and during that meeting, asked for my participation in a restorative practices process structured around 6 questions for all participants: What happened? What were you thinking at the time? What have you thought about since? What impact has this incident had on you? What has been the hardest thing for you? What do you think can make things right? The idea was that after separate moderated dialogues with various parties addressing these questions, there would eventually be a moderated conference discussion between the parties. Acknowledging the pain and distress within the community, and the need for clarification of my actual views, I agreed to participate.

During the week of March 27–31, the HSPH administration sent out a series of e-mails. The three pieces described above were collectively referred to as “VanderWeele's writings on gender identity, marriage, and abortion.” My “writings” consisted of about 1300 words (I was not an author of the *amicus* brief, but rather one of 47 signatories; the brief itself was itself one of 149 such briefs in the case, 77 supporting the petitioners, 67 supporting the respondents, and 5 supporting neither). *E*-mails went out from the Dean and Academic Dean to the Department Chairs and the School's Academic Council; from the Dean of Education and the Directors of the PHS PhD Program to the students; and from the Chief Diversity, Inclusion, and Belonging Officer and Dean of Education to Epidemiology students. The e-mails noted that some students were feeling harm and betrayal, and that eight 2-hour circle dialogue listening sessions would be held as part of the restorative practices (meetings that were to take place without me, so as to hear the concerns of students, staff, and faculty). The e-mail to the department chairs indicated that the chairs should meet with their faculty to discuss the matter. Following these e-mails, I asked to meet with our Dean and Academic Dean. During that meeting and through e-mail correspondence I indicated that I was indeed prepared to participate in the restorative practices. I also requested that students be reminded of Harvard's commitment to the principles of academic freedom (e.g. [8]) and of the absence of any academic misconduct on my part. I also sent the letter that the Stanford Law School Dean had written on principles of academic freedom [9], following the turmoil that had just occurred there. The HSPH Academic Dean at least found the letter “very compelling.” I proposed various other approaches to promote civil discourse and intellectual diversity within HSPH. The Deans indicated they would consider the proposals.

Several further e-mails were sent out by Department chairs and Program Directors. Some of these e-mails referred to my views as “reprehensible”, as being such as to “cause deep hurt, undermine the culture of belonging, and make other members of the community feel less free and less safe,” as having been “condemned” by “many students, faculty and staff,” and as “in conflict with our Department's and the School's stated goals of advancing Equity, Diversity, Inclusion, and Belonging as well as our commitment to sound public health policy,” with the incident itself described as an “extremely corrosive situation,” and the restorative practices as “redress” and “reparative justice.” These e-mails were sent to large listservs of students, faculty, and staff. Following two of the more strongly worded e-mails, I wrote to the respective department chair and PhD program director. In one instance I received a kind and apologetic note, followed by a public apology to the entire department. In the other case, the language that was used was defended. None of these various e-mails made any reference to the absence of academic misconduct, or that my writings were protected by Harvard's policies on freedom of expression.

Perhaps in part because of this lack of clarity, the situation continued to escalate. Students raised concerns with faculty in courses unconnected to these issues. It was clear that there was deep pain or a feeling of being offended among many within the HSPH community, sometimes over perceived threats to identity. Some of this seemed to be that the incident and subsequent discussion had allowed a long series of past hurts and harms within the LGBTQ+ community to resurface. Some of it seemed to be a sense from members of the LGBTQ+ community that what they had thought they had come to as a “safe environment” was in fact not so. There was perhaps also a sense that my writings had violated HSPH community norms and values. This is a complex matter, which I will return to later, insofar as the values, norms, and systems of moral understanding present within HSPH are somewhat less uniform than the School often projects, though the majority positions on these issues are indeed quite clear.

Throughout this time, I agreed to every request from faculty, staff, or students to meet, either individually or in small groups, to talk through the various issues, or for them to share their pain. Faculty, including the Epidemiology Department chair, who defended me, or the principles of academic freedom, sometimes themselves came under criticism. Several faculty and students, including some who strongly disagreed with my views, nevertheless wrote to affirm support, and the importance of the free exchange of ideas. It also became apparent that a number of students and some faculty agreed with my views, but felt silenced by, and concerned about, what was taking place. Students also expressed concern that the way the circle dialogues were being handled suppressed alternative viewpoints.

Some students and faculty expressed the view that even if I did have the right to academic freedom, it was nevertheless problematic that I had signed the *amicus* brief with my academic affiliation. I tried to clarify with faculty and students that (i) the brief itself stated that affiliations were for purposes of identification only; (ii) this was in line with Harvard policy ([35], Section II.2); (iii) this was standard practice for academics signing briefs. Some faculty (including those involved in the national movement to protect academic freedom) expressed concern about the restorative practices, and advised me not to participate, especially in light of the fact that the administration had not clarified that there had been no misconduct, had not affirmed principles of academic freedom to students, and that words like “redress” and “reparative justice” had been used in some of the e-mails. I defended the restorative practices process, provided that proper clarification was given, on the grounds that its framing in terms of the six questions above was reasonable, and that it was important for all parties to seek the restoration of relationships and trust.

At the Epidemiology departmental faculty meeting on April 5th, a central agenda item was “Discussion on matters related to Tyler VanderWeele's views.” The Department chair allowed me to make some remarks prior to this discussion. I commented that I had real sorrow over the pain and distress in the community; that my view at the time of my signing was similar to that President Obama held upon his election and until 2012; that I had been sent the *amicus* brief, asked if it corresponded to my views and, if so, if I were willing to sign. Since it did correspond to my views, as a member of our democracy and as a matter of conscience, I thought it was important to sign. However, I further noted that, as a member of that democracy, I had also accepted that a different view of marriage had prevailed in law, and that I had not addressed the matter since. I noted that I worked hard to treat all students respectfully. I also said that my experience of the events made me feel that HSPH, as a community, was not particularly strong on dealing with matters of academic freedom, intellectual diversity, and civil discourse. After my remarks, I departed from the meeting to allow for freer discussion among the faculty.

During the week of April 10–14, six more 2-hour circle dialogue listening sessions were scheduled. I again met with the Dean who affirmed my academic freedom, but defended a decentralized approach to the incident so as not to upset the students and so as to let the situation quiet down. There was also to be a transition of Deans in the new academic year and I was told further work on supporting academic freedom would likely take place then. The University's Vice-Provost wrote to me stating, “I know I speak for all in the University's administration when I write that we respect you and your opinions, and your rights to free expression.” I subsequently met with her and she affirmed the same. On April 14th, I requested that the Deans communicate to the Department chairs both the University-Wide Statement on Rights and Responsibilities concerning free speech [8], and also that, as per the comments above, my signing with my academic affiliation was within bounds of Harvard policy ([35], Section II.2), and that the chairs then distribute this material to the faculty who could then clarify matters with the students. I argued that this would be in keeping with her proposed decentralized approach.

A week later, on April 21st, I was notified by the Dean that there was to be a cessation of scheduling additional circle dialogues, after the fourteen 2-hour sessions that had occurred; and that the University-wide statement on academic freedom, and University policies on using one's affiliation, had been distributed the Academic Council and Department chairs; the e-mail also expressed an expectation that in the new academic year there would be additional teaching and learning modules on academic freedom. To the best of my knowledge, however, no departmental-level communication and clarification was made to either faculty or students during the week that followed, or thereafter.

In May, a faculty colleague mentioned that she had sent one of my statistical methodology papers to a collaborator, and that it had been dismissed because of my signing the *amicus* brief. It seems there were similar dismissals of my methodological work on such grounds on Twitter, and by some HSPH students. Throughout this time, I felt uncomfortable entering the HSPH buildings or using my office there, and only in early May did I return. I had previously spoken with several people who had said that they were uncertain whether, if I entered, there would be organized attempts to surround me. In the final week of the semester, I attended a major departmental event to move towards greater re-integration, and also participated in discussions with PHS doctoral students, facilitated by the Associate Director of the PHS program, to better understand different experiences and viewpoints concerning marriage, rights, and other moral questions. On May 8th, nine days before the date set for the formal concluding joint restorative practices conference, the moderator of that conference informed me that the final two of the framing questions, “What has been the hardest thing for you? What do you think can make things right?” would only be asked of the other participants, not of me. This created an asymmetry in the process, effectively considering the feelings of hurt, and placing blame for the situation, in only one direction, thereby arguably reinforcing the concerns of my faculty colleagues who suggested that I withdraw. Nevertheless, in hope of some relational restoration, I decided to see the process through. I certainly do not think faculty with unpopular minority viewpoints should be subjected to this, nor do I feel I was forced to participate, but out of desire to engage with those who felt most affected by what had taken place I went ahead. The spring semester of the academic year concluded without any formal clarification from the administration to students along the lines I had requested.

The message that, to my mind, was implicitly conveyed by the administration to the HSPH community, often by way of innuendo and what was not said, was that my views either perhaps were not, or perhaps ought not be, protected by academic freedom.

## 3 An analysis of the response

Throughout March and April, I was often spending six or more hours per day dealing with the matters above. However, little of that time was spent in clarification of, or discussion of, my views. Almost all of it was devoted to managing the situation. I learned later that some thought my views constituted a threat to human rights. I have, in the Appendix of this essay, tried to provide greater clarification of my viewpoints on each of the three written pieces. This seems important in considering the question of what people and viewpoints should be considered admissible in academic public health, which I will turn to in the next section. In this section, I would like to address aspects of the response to the events that I think were not conducive to academic life within a university context. I will offer my interpretation of the events as I experienced them, though I certainly acknowledge that others may well interpret them differently and that there are undoubtedly numerous details of the events themselves concerning which I am not aware.

In almost all cases, I do not think the actions of the HSPH administrators were ill-intentioned. Essentially all of my interactions with the Deans, my Department Chair, and the Chief Diversity, Equity, and Inclusion Officer were interpersonally positive and supportive, and I am grateful for this. I would also speculate that these events would likely have played out similarly, at perhaps not all, but at most, other schools of public health in the United States. I certainly do believe that students, staff, and faculty have every right, as part of their own freedom of expression, to be upset about, and to criticize, my published writings. However, I believe the way that this was handled by the administration, or in some cases by faculty or students, has detracted from academic life within the community.

First, although the administration acknowledged that my opinions were protected by freedom of expression and that I had not committed any academic misconduct, they seemed unwilling to formally communicate this to students, staff, or faculty. I proposed several different ways the clarification could be made –a letter from the Deans, communications from Department chairs, clarity by the Chief Diversity, Inclusion, and Belonging Officer– but these points were never publicly made to the HSPH community. Without having the necessary clarification, students requested that my tenure be revoked and that I be fired, or that I be removed from my teaching position, requests that would be unlawful for the School to carry out.

Second and relatedly, I was told that some of the students stated that my signing the *amicus* brief should not be protected at all. This is tantamount to denying my right to participate in our democracy. The School not affirming that my signing was protected perpetuates such beliefs.

Third, the two instances of which I am aware in which a department chair or PhD Program Director publicly condemned my views to an entire department or program, in their role as University administrators, constitute violations of Harvard's University-wide Statement on Rights and Responsibilities [8].

Fourth, the lack of clarification also had a chilling effect on freedom of expression of others. Some students expressed concern about there being a double standard on freedom of speech. They felt that members of the HSPH community were only free to hold and express opinions so long as they aligned with the vocal members of the academy. If this was how a tenured professor was being treated for occasionally writing about his views, what would happen to an untenured professor, or a postdoc, or a student?

Fifth, in many cases, there seemed to be condemnation of my views before inquiry and understanding, not only from students but also from a department chair. Certainly not in all, but in some cases, the logic seemed to be that since the political case was won, the intellectual case must also be considered settled, and that one could thus condemn. This likewise does not facilitate an environment conducive to the free exchange of ideas.

Sixth, for a number of people, there seemed to be a reliance on information from social media posts, rather than a reading of the actual documents. The Twitter posts reached over 40,000 individuals within a few days. While the conversations I have had I think have been helpful in clarifying viewpoints, and sometimes in the restoration of relationships, I simply cannot meet individually with 400, or 40,000, persons. I do not think anything of the scale of what took place would have been possible without Twitter, which seems to now exert undue and unhealthy influence on academic discourse. The Twitter posts suggested that I was homophobic, racist, and unfit to study flourishing. (For what it is worth, I am open to, and have, friendships with people across a diverse range of ideological viewpoints and identities, and value these friendships both in and of themselves and with regard to what I learn from them; it is also the case that there are a range of perspectives on the above controversial issues within the Human Flourishing Program at Harvard that I direct, and I welcome that diversity). Sadly, these ad hominem accusations were perpetuated by academics – my colleagues in public health. Unfortunately, the abortion and mental health commentary was behind a paywall, as are all *JAMA* Network commentaries, and this allowed those who published the Twitter posts to make various innuendos as to the content of the commentary, without the commentary being read or easily accessible. The HSPH administration likewise did not distribute the actual article, but instead linked to the website behind the paywall.

Seventh, even when the pieces were read, the judgements that were made were often on my implied or assumed views, rather than on what was actually written. It was surprising how quickly people would sometimes jump to conclusions about my beliefs concerning positions that I simply do not hold. Such practices of guessing, assuming, scrutinizing, and condemning someone's unstated views seem inappropriate. Such practices I think also tend to lead to a greater propensity to attack the person, rather than the ideas.

Eighth, there was effectively no attempt to properly attribute responsibility for the pain within the HSPH community. Certainly, I signed the *amicus* brief and for that I take full responsibility. However, I was not myself seeking to disseminate either the brief, or my views on this matter. With regard to the law, I had accepted the outcome of the Obergefell vs. Hodges Supreme Court case. Others may see things differently, but from my perspective, for some group, possibly involving members of HSPH, there was an attempt to use my signing of the brief to harm my professional reputation, which was apparently more important to them than the wellbeing of the HSPH LGBTQ+ students, my own well-being, and the fabric of the School community.

Ninth, lack of public affirmation for academic freedom will inhibit empirical research being carried out from those with diverse viewpoints. Beyond the concerns noted over my signing the *amicus* brief with my academic affiliation, there were additional concerns over my signing without having carried out original empirical research on the topic. While the topics of the *amicus* brief were not, and are not, my primary topic of research, I had read through much of the related empirical literature through 2015 (though I have not closely followed developments since), and I had provided critical feedback on a review of that literature, mostly concerning methodological critique of study designs. This work, along with my carrying out related conceptual, philosophical, and cultural readings on the topic, which put me in a position to reasonably sign the brief, were an exercise of academic freedom, though the signing itself was an act of freedom of expression, arguably protected by academic freedom [13,14]. I have in fact additionally been invited to participate in original empirical research on each of the three controversial topics mentioned above. I have declined all of those invitations. In an academic environment more supportive of free inquiry I may well have pursued one or more of the aforementioned requests. While I think high-quality research is important on these topics, and should be protected by academic freedom, and I believe I could have contributed through my methodological expertise as I do on many other topics, the professional hazard in the current environment seemed too high. My experience with the remarks that I have made on these issues seems to have validated my prior concerns.

Finally, the way this was handled consumed an enormous amount of time, not only for me, but for many in the HSPH community. I accept that actions have consequences, but those consequences depend also on the response of the community, and the academic health of that community. An alternative for those who disagreed with me would have been to respond with, “I believe his viewpoint is wrong, and I am glad that the position that he was defending lost,” and then either move on, or otherwise engage with me individually, or in groups, on the viewpoints themselves. That my positions were not simply treated as minority, though potentially intellectually defensible, viewpoints, but were instead effectively broadcast as being problematic (with the types of expressions described above) to what I believe was nine departments and perhaps up to over a thousand students, faculty, and staff, very much altered, for me and others, the amount of time required to move forward, the nature of the exchanges that took place, and the understanding of the institution. As noted above, in spite of all of the time spent, for nearly two months there was remarkably little exchange of ideas, or trying to understand diverse viewpoints, and learn from one another.

To my mind these aspects of the response to my writings do not constitute, nor do they foster, a healthy academic community. Until the leadership and administration publicly and actively affirm and defend academic freedom and freedom of expression, incidents of the sort that I have experienced will inhibit the free exchange of ideas, understanding, and the pursuit of knowledge.

## 4 Academic public health

The challenges to academic freedom in this case were somewhat convoluted. The Vice-Provost, the Dean, and my Department Chair all affirmed my freedom of expression, but there was a reluctance on the part of the School's leadership to *publicly* acknowledge this. The message that I felt was often being conveyed *to me* was that my views, while perhaps formally protected, should not in fact be present within academic public health. Fundamentally, I think there is a lack of respect for the intellectual diversity within our public health community. In this section, I will argue that academic freedom and freedom of expression need to be supported to create a respect for viewpoint diversity, and this diversity, when engaged with through rational civil discourse, has tremendous value for knowledge and understanding, for societal engagement, and for population health.

I believe much of what occurred took place because of different systems of moral understanding within the School. The majority positions at HSPH on abortion, marriage, and gender identity are relatively clear. My views, occasional writings, and signing the *amicus* brief were seen by some as violating the norms and values of the School. It is the case that I am a Catholic, and the positions that I hold follow the teachings of the Catholic Church [17]. I have not in any way hidden my Catholic faith; indeed my being received within the Catholic Church was described in the HSPH Magazine [40]. I assume Harvard faculty members are allowed to be Catholic, and that Harvard is not supposed to discriminate in the treatment of its members on the basis of religion. However, in the legal counsel I have received, there were questions as to whether, in the administration's behavior towards me, Harvard is meeting those obligations to not thus discriminate. With respect to the three issues that caused controversy, although my views follow the teachings of the Catholic Church [17], it is also the case that Catholic teaching is that various moral positions can also be derived on the grounds of reason [43]. I believe it is good to uncover and make use of those grounds so as to present more generally accessible arguments. In my engagement with these issues, I have put forward arguments that are accessible in a secular context. Although I do not think it is necessary to do so, I tend to think that a democracy functions best when the grounds of the arguments put forward are accessible to as broad a group as possible. Similar viewpoints to mine are also held by many others, on, or apart from, the grounds of faith.

A couple of years ago, a student asked me how I could survive as a committed Catholic at an institution like HSPH. My response then was that this had not been an issue for me; that I realized that the vast majority of the faculty disagreed with a number of my views, but this hadn't inhibited my work, and, on the whole, the School had been a supportive environment. My perspective on this question is now rather different. The student's question, and my experience these past months, raise further concerns regarding who is welcome to participate in academic public health, and in what manner.

A university ought to be a place in which a broad range of viewpoints are welcome, even those which may be strongly at odds with one another [6,67]. The members of an academic community have a responsibility to put forward reasoned arguments, but we come to various topics with different starting points and presuppositions. The process of rational discourse is in part meant to uncover those presuppositions, and to evaluate the extent to which logic and evidence support a given conclusion. All research and scholarship – my own and others – is influenced by a person's commitments, identities, and positions. By interacting with those of other viewpoints we are made better aware of those influences and are together better able to try to discern truth. We should conduct our discussions and arguments respectfully, with the recognition that others will often disagree with us and may do so passionately. Through civil discussion, our understanding of alternative viewpoints becomes stronger. Our understanding of our own views can often also be sharpened; and we can sometimes find common ground. Civil discourse and viewpoint diversity are the means; knowledge and understanding are the ends.

Not everyone may agree with these ideas. Some may view the notion of rational discourse as one of many attempts to seize power. Others may disparage the notion of respectful civil discourse. One thread of the Twitter posts put forward the notion that my being “fastidiously interpersonally kind” was itself potentially problematic in that “fastidiously interpersonally kind oppression” is common in spaces of privilege. The reaction of some people to my abortion and mental health commentary was to reject the notion that there is potential common ground for those on different sides.

From the perspective of public health advocacy with a particular agenda, these alternative viewpoints are themselves perhaps understandable. An approach which rejects reasoned engagement, civil discourse, and finding common ground may sometimes be the fastest way to one's end. But, as discussed further below, I do not think it provides much hope for the future of a pluralistic democracy. Moreover, such an approach detracts from a university's purpose to create, preserve, and disseminate knowledge; it instead alters that purpose for different political ends.

These issues raise the question as to whether a school of public health, situated in a university, is, or should be, more akin to a public health advocacy organization, or to a university of the nature described above? The answer to that question is what is at stake with regard to the events at HSPH, and how the situation was handled by the administration. If HSPH is viewed principally as a public health advocacy organization with a particular agenda, then I have violated community norms and values and some form of redress seems necessary. If HSPH is viewed principally as an academic institution, then my views, provided I can defend them, should be a welcome part of dialogue, allowing for deeper understanding of one another's viewpoints.

There has been, and likely always will be, a dual nature to academic schools of public health. They function both as academic units, and as public health advocacy organizations. But decisions need to be made as to how to treat their members. Can the viewpoints and convictions of a Catholic who is faithful to the teachings of the Church, or an Orthodox Jew, or a devout Muslim in an analogous situation, or a conservative, or others, be openly expressed, and that person still be treated civilly? If the answer is no, then there is a real loss with respect to the School's academic nature. If the answer is yes, then I think we have a long way to go.

There needs to be greater clarity as to what views are admissible in public health discussions, and which are to be considered unacceptable. Should it be permissible, for example, to silence or exclude minority viewpoints that are held by 10% or 30% or nearly 50% of the American population? Clarity on such issues would help address the question of the extent to which the university considers it acceptable for me to share my viewpoints on moral controversies, or to carry out related empirical research, or both, or neither. The answer to these and related questions are not at present entirely clear at Harvard [3]. Beyond the question of what views are admissible, there is an additional question as to whether diverse viewpoints should in fact be sought out. The research at many schools of public health is predominantly supported by federal grants, publicly funded by taxpayers. To what extent should the diversity of viewpoints within the general public not be only permitted, but even actively represented, within academic public health?

These concerns are not merely academic or theoretical. Schools of public health train and shape our nation's future leaders. On the various controversial issues noted above, roughly 30% to 50% (or more) of the United States' population hold positions similar to my own [29,51]. Such groups thus constitute 100 million or more people, just in the United States. There is not the same distribution in viewpoints within this country (or the rest of the world) as one finds at HSPH. To what extent are we equipping future public health leaders and academics to deal with this diversity of viewpoints? To what extent are we providing an environment in which to even *understand* different viewpoints?

Encounter with diverse viewpoints can be challenging and threatening; and indeed there were claims that students did not feel safe. That all students *are* safe is critical; that all students *feel* safe seems beyond the capacity of any institution, and making such feelings a central goal is likely to compromise learning. Excessive protection from ideas and people with whom one disagrees can make a person weaker emotionally and psychologically [4], weaker in understanding and knowledge, less able to find common ground, and less able to serve the entirety of one's country and world. If public health becomes, and is viewed as, overly partisan –as not even capable of understanding the concerns of others– then trust in public health institutions will likely continue to erode. This, I believe, will often gravely compromise the capacity of these institutions to promote population health.

Freedom of expression can be abused and there are risks to granting these freedoms [71], but by treating one another civilly and respectfully we can and should try to prevent those abuses. There are also complexities around differentials in power within the university, and the reach that the speech of a particular person is able to have, though I believe that most faculty are genuinely motivated to try to empower students so that they too, as their career develops, have the capacity to communicate their work also to the general public. However, without taking the risk of guaranteeing freedom of expression for everyone, as best we can, there is potentially a severe loss with regard to our own capacity to seek truth, and also a severe danger with regard to our capacity to work together towards promoting well-being. The loss is well-characterized by John Stuart Mill, in his work *On Liberty* [54]: “He who knows only his own side of the case knows little of that. His reasons may be good, and no one may have been able to refute them. But if he is equally unable to refute the reasons on the opposite side, if he does not so much as know what they are, he has no ground for preferring either opinion…” The danger is perhaps well-characterized in an address of Frederick Douglass [25], delivered in Boston, not far from HSPH: “Liberty is meaningless where the right to utter one's thoughts and opinions has ceased to exist. That, of all rights, is the dread of tyrants. It is the right which they first of all strike down.”

The only way that we can have true inclusion and belonging for everyone is a radical openness to the free exchange of ideas, carried out respectfully and civilly, accepting that others will disagree with us, accepting that we have different moral understandings about right and wrong, and accepting that we may find some ideas painful and hurtful. Moral understandings are diverse, and most nontrivial ideas about policy will likely be hurtful or offensive to at least some. Many students found my signing the *amicus* brief hurtful, and many likely view my signing as morally wrong. Conversely, I likewise view advocacy aimed at intentionally increasing abortions as hurtful and morally wrong. However, both of these actions are protected within our constitutional order, and are also within the bounds of academic freedom. Our democracy and universities should be able to sustain such diversity and disagreement. This does not mean that various moral views, or values, or identities shouldn't come under scrutiny. On the contrary, I think there should be open disclosure and debate of moral systems, values, identities, and their grounds, including religious grounds. This again allows for a better understanding of others' and our own perspectives, and also opportunities both for reasoned persuasion and for finding common ground.

The alternative for academic public health to a more radical openness to a free exchange of ideas is to exclude, or silence, or suppress, alternative viewpoints. One might take the position that Christians, Jews, Muslims, conservatives, and others are welcome so long as they in fact agree with the majority viewpoint, or remain silent on certain issues. That may work, and perhaps to some extent has worked, at schools of public health. However, it is not similarly an option for our society. Within society, it seems that we are faced with only the options of increasingly vitriolic fighting, or alternatively of attempting greater civil discourse, attempting to find common ground among our pluralistic perspectives, and accepting that the democratic process will sometimes not turn out as we like. Without that acceptance, polarization and hatred are likely to continue to increase. The question then arguably arises as to which of these two approaches to society will schools of public health ultimately contribute. The relative balance of its contributions could make a great deal of difference for the future and well-being of our democracy.

We need a robust free exchange of diverse viewpoints so that we can engage civilly and thoughtfully in society. Civil discourse need not exclude the expression of anger over offense. However, anger and hurt do not in general entail a right to silence the speech of others [6,27]; nor do they constitute a refutation of rationally grounded arguments; nor do experiences of anger and hurt necessarily entail evidence of wrongdoing or injustice. While we can acknowledge anger and hurt, we also need to make these principles of discourse clear to one another, and to train ourselves to be able to engage with those with whom we disagree even amidst anger. Moreover, it also needs to be acknowledged that, due to differing values and presuppositions, there will often be anger and hurt that extend in both directions. Such recognition can again foster a freer exchange of ideas, even amidst passionately held views. Among the proposals I made to our Deans were the following: (i) implementation of further training on the positive value of academic freedom and the free exchange of ideas; (ii) regular data collection on whether students, staff, and faculty feel comfortable sharing what they really think about controversial issues, both inside and outside of the classroom; and (iii) the introduction of a new seminar series on understanding diverse intellectual viewpoints, which would bring together two speakers on different sides of an issue to model civil discourse, to help us uncover differing presuppositions and values, and to hopefully find common ground. These practical steps, among others, would help foster a healthier academic community, and one more respectful of intellectual diversity. I believe there would be benefit from adopting such practices throughout all schools of public health.

Different communities – whether LGBTQ+ communities, or different religious communities, or different political communities – will have different values, and different understandings of what is good. Questions concerning means and the efficacy of policies can, to a certain extent, be addressed by empirical research. But questions concerning values, and the nature of well-being, cannot. Within a pluralistic society, we can try to structure life and policies so each of our distinctive communities is empowered to try to also pursue the values and ends that they deem most important. These distinctive values will, however, inevitably sometimes come into conflict. Our democratic system provides a way to adjudicate between differing viewpoints. However, there also needs to be a realism as to what political action will, and will not, accomplish. A policy or change in law can of course grant new freedoms, and rights, and responsibilities, and can restrain or enable action and behavior in various ways. However, its effects on beliefs and values are more complex. Policy and law will influence beliefs and values, but law cannot force such change, and it will often not alter the beliefs and values of a particular community. Shame has sometimes been used to try to bring about such alterations, and this can sometimes be effective in altering more loosely held values and beliefs. But it can also be resented, and it sometimes only alters what people are willing to say they believe, rather than what they actually believe. Moreover, shame is less likely to alter values and beliefs that are firmly held and rationally grounded, or values embedded within a community's life. For those to change, rational discourse and persuasion, as well as consideration of a community's lived experience, are ultimately needed.

An overemphasis and focus on our disagreements, which to my mind is what much of the culture wars have brought us, will lead to greater conflict. It is not that these disagreements do not matter – they do matter – but there is a question as to how much emphasis they are given. Are they the central focus of our political energies, or are these important but auxiliary topics with respect to our interactions with others, and a source of genuine mutual respectful acknowledgement that we do not agree on all things? Through civil discourse and a free exchange of ideas we can understand each other's values and notions of well-being more fully. We can come to understand that reasonable people of goodwill can disagree on important matters. We can also see where there might be common values. I have argued elsewhere that such common values extend to a number of aspects of flourishing including happiness, health, meaning, character strengths, relationships, and financial stability; and that we can meaningfully work together to pursue policies that promote various aspects of flourishing held in common [7,[76], [81]]. This is what much of the work of the Human Flourishing Program at Harvard is trying to accomplish (and is also where I try to focus my own energies; though when controversial issues are presented to me, I will continue to speak my mind, as I hope will others regardless of their viewpoints). I truly believe that a healthier more robust free exchange of ideas, values, and viewpoints, carried out civilly, has the capacity to highlight our agreements and common pursuits, and to respectfully acknowledge and try to navigate our disagreements. Academic institutions should view the advancement of skills to work together, across differences in moral systems, values, and identities as a critical part of preparing leaders and academics to promote the common good. My colleagues certainly might see a number of these issues differently and I would welcome them to share their alternative perspectives.

I conclude with discussion of the relation of these issues to a few other specific aspects of my own life and work – past, present, and future. During the 2004–2005 academic year, when I was a doctoral student at HSPH and serving as President of the Student Christian Fellowship at the School, a member of that fellowship indicated a desire to start a pro-life group. With some fear and trepidation, I agreed to help her. The School administration was in fact supportive. When posters advertising events were pulled down, the School put them behind glass. In the end, we hosted joint events with the Student Reproductive Rights group at HSPH, both to better understand each others' perspectives and also to try to find common ground. It is not clear to what extent we are positioned to hold similar joint undertakings today.

One can, in one's discussions, at least still point towards examples of partnerships navigating disagreements. In the elective course I teach at HSPH on religion and public health [82], I discuss the partnership between Brazil's National AIDS Program and the Catholic Church [57]. That partnership persisted, in spite of deep and irreconcilable disagreements, including highly pertinent ones regarding advertisements for contraception, because both groups believed they could better advance their shared goals by working together than by working separately. That sort of difficult partnership could be taken as a model as to how to move forward towards common ends, even when there is deep disagreement over values. There are of course numerous other such examples [16,33,41,42,48]. However, without this sort of difficult work together, I think progress towards societal well-being will be impeded. There are approximately 2.4 billion Christians world-wide, 1.9 billion Muslims, and billions of people of other faiths [61]. Their viewpoints are diverse, but many hold positions similar to the positions I hold that were found problematic at HSPH. Schools of public health have the option of working to oppose, suppress, and silence those views; or may hope to change or convert their views; or may acknowledge the disagreements and nevertheless find ways to work together in our various societies across the globe. The distribution of views of academics within schools of public health on the three issues above are not representative of the diversity one finds worldwide, and it is not clear that this is likely to change. Some projections suggest that the proportion globally who identify as religious will increase over the coming decades [62]. It seems worthwhile to have conversation on what might be the best way forward.

I will, next year, be publishing a book entitled *A Theology of Health* [81], from a distinctively Catholic perspective. Had it not been for the events that were ignited by the Twitter posts, it is possible that the book would have mostly slipped under the radar of the public health community. With the events that have taken place these past months, the book may now come under much greater scrutiny. While the book lays out a distinctively Catholic understanding of health, it also engages with the empirical literature, and it concludes with a “non-theological post-script” which attempts to bring some of the insights of a Catholic or Christian understanding of health to a more pluralistic context. I am sure that there will be critique, and in fact, I welcome it. But I hope that the criticism will be productive, that it will allow me to understand the views of others, and challenge mine in helpful ways. I likewise hope the book provides similar opportunities to others to have a better understanding of my views, and to have their views challenged and sharpened; and that ultimately it may help us work together.

The events recounted above at HSPH also happened to coincide with two other significant undertakings. First, in the fall of 2022, long before I had any idea these issues would arise so personally for me, the Associate Director of the Human Flourishing Program at Harvard which I direct, began to help organize a faculty-led Council on Academic Freedom. He had my full participation and support, though he began this work on his own initiative. That Council was formally constituted in March of 2023 [63], not long after the Twitter posts began. Part of the mission of the Council is to support faculty attacked for speech. Given my early involvement in the Council's formation, I did not, however, particularly want to be the Council's first case. I have received helpful advice from the Council's co-Presidents, but asked them not to collectively act until the beginning of the new academic year. My hope is that over the long-term, the Council will be able to help strengthen the School with regard to dealing with intellectual diversity, civil discourse, and freedom of expression. I would invite other faculty at HSPH, and within the broader Harvard community, to become members and join in these efforts [22].

Second, in April of 2023, the Human Flourishing Program, in collaboration with Harvard's Memorial Church and other organizations, hosted a conference on forgiveness that had been over a year in planning. I see forgiveness as replacing ill-will towards someone you believe has harmed or wronged you with goodwill [38,77]. There is perhaps a sense of moral injury, both for me, and for those who feel hurt by my writings and action. For them, it may be partially constituted by my holding a view of traditional marriage that they feel threatens identity and human rights, and of HSPH not being the community for which they had hoped. For me, it is partially the attitude my colleagues in public health now seem to have towards me, and partially being within an institution, and perhaps a discipline, in which my views seem not to be welcome, in what I had previously understood as a principally academic context. My *Psychology Today* blog post on the topics of forgiveness [80], the conference, and our randomized forgiveness workbook trial [37,88], was strongly shaped by what had been taking place at HSPH. I recognize that there is real pain on both sides, and that we each view the others' actions as having been harmful. I recognize the challenge of forgiveness when one or both sides believe they have not done anything wrong. While I hold my views with conviction, I have genuine sorrow for the pain and distress within the HSPH community and I hope that the various discussions that have taken place eventually bring greater restoration of relationships and trust. Forgiveness is not sufficient for healing, restoration, and rebuilding; one needs also understanding, accountability, mourning over loss, and new ways forward; however, I do think that forgiveness – replacing ill-will with good-will – helps move in this direction. I have been working towards forgiveness of those who have, either unintentionally or intentionally, hurt me and my family. While, from my perspective, I believe I have done no wrong in holding or acting upon my views, I acknowledge that others see things differently, and I hope, over time, they too, from their perspective, might see forgiveness as an appropriate response. I believe that through civil discourse, and through forgiveness, we can bring some healing to our HSPH community and I hope also, in the long-run, to our world.

## Declaration of Competing Interest

The authors declare that they have no known competing financial interests or personal relationships that could have appeared to influence the work reported in this paper.

## Appendix A Moral controversies and clarification of views

In this Appendix, I would like to try to further clarify my views concerning each of the three written pieces that caused controversy. This seems important both to aid understanding of minority viewpoints in academic public health, and to serve as a potential case study as to the question of what views should be considered admissible within the public health community.

Before turning to each of the three pieces, I would like to put forward a series of positive statements to help put my positions in the three written pieces within a broader context: (i) I believe all people are of equal dignity and worth, (ii) I believe all people should be treated civilly and with respect; (iii) I believe that, on account of their shared humanity, all people have equal human rights that should be respected (though there may be disagreement as to what those rights are); (iv) I desire the well-being of all people; (v) on various controversial political issues, I hold some views that are, at the present time, in the United States, currently associated with the left; and I hold other views that are, at the present time, in the United States, currently associated with the right; for me, the most important question on each issue is not political allegiance, but on how policy relates to societal well-being; (vi) while I do have views as to how best to bring about well-being at the societal level, rather than just the individual level, I know that others may strongly disagree with those views, and I respect their right to disagree; (vii) I am committed to our democratic process as the means to adjudicate between different views on policy and law, and fully accept that this has and will often lead to policies different from what I think best.

I will now consider each of the three written pieces in turn. The position put forward in the *amicus* brief was that what was at stake concerned the *definition* of marriage. I believe that the traditional definition of marriage may be construed as something along the lines of “a vow of permanent union between a man and a woman.” This definition ties marriage to children in two ways: first, that it is between a man and a woman ties the definition directly to reproduction (a sperm and an egg are still necessary for reproduction); second, the vow of permanent union creates a stable environment for children. Marriage is both about the emotional, relational, and sexual bond between spouses, and also about children. It is not that children are present in all marriages, but that marriage, as an institution, supports both children and spouses, and their interconnection. It serves dual related purposes. I believe that there are at least two ways in which this definition can be modified while still retaining something meaningful. One might alter the “vow of permanent union” or alter its being “between a man and a woman.” I believe these two alterations are in principle independent of one another. The first alteration gained greater cultural prominence with no-fault divorce; the second with growing acceptance of same-sex marriage. With both alterations in place the definition of marriage becomes something along the lines of “an expression of intention of long-term union so long as the partners involved desire it.” However, once again, the two alterations are in principle independent; same-sex partners may make vows of permanent union; conversely, many opposite-sex weddings today do not involve vows of permanent union. Even with both alterations in place, this is still a meaningful understanding of marriage, but it places more exclusive emphasis on the personal fulfillment of spouses, and it severs the direct connection of marriage with reproduction and children. It does not exclude it, but it becomes a secondary, rather than a co-primary, purpose of marriage.

The argument of the *amicus* brief was that since what was at stake was the definition of marriage, it seemed better to allow the American people to deliberate about the definition through a democratic process within each state than to entrust the decision to nine unelected Supreme Court justices. There is certainly no guarantee that the democratic process within states will necessarily be representative either, but it does in general seem more feasible to attain a more substantial consensus on many issues at the state level than at the federal level. In any case, with the Supreme Court decision, the latter definition effectively prevailed in law. With marriage thus redefined, I can affirm a “right to marry,” with marriage thus understood, but once again, my view was that with respect to overall societal well-being, it would have been better to use the term “civil union” for the latter definition and to retain the traditional definition of “marriage” itself. However, I respect that others disagree with that position. In any case, in whatever way marriage is defined, each person has a right to enter marriage, though with the traditional definition, it would be unusual, though not inconceivable, for a same-sex attracted person to marry under that understanding. Understandings and definitions of marriage change over time [36]. Our dominant cultural understanding has considerably evolved, but I do not believe that the redefinition of marriage is without impact on the welfare of children. Marriage has not been the only institution oriented towards the welfare of children – education is as well – but marriage has been a primary one. The redefinition does not completely sever the link with children, but it subordinates it. That the redefinition takes one of the primary institutions oriented towards the welfare of children and re-orients more exclusively to the fulfillment of spouses I believe has a number of consequences.

First, as argued in the brief, the traditional definition of marriage best protects Article 7 of the United Nations' Convention on the Rights of the Child [74] that the child shall have, “as far as possible, the right to know and be cared for by his or her parents.” I believe this proposed right is directly connected to childhood well-being [21,45,46,52,53,55,60,64,84,85], as are other parts of the Convention's statement. This point also in some sense subsumes the point in the brief concerning sex-complementarity within marriage, since children living with their biological parents would naturally entail such complementarity (though, of course, even independent of the question of same-sex marriage, this will not always be attained in the raising of children as, for instance, with single parents, divorce, widows/widowers, or some cases of adoption).

Second, a revised definition of marriage, more disconnected from children, also alters the understanding of opposite-sex marriage as well. If marriage is principally about the fulfillment of spouses, this will then alter a person's willingness to stay in a marriage for the sake of the children. For a number of people, it will no longer make sense to continue with a marriage if the marriage itself is not personally fulfilling. However, the impact of this on children is not inconsiderable. With an altered understanding of marriage, focused on fulfillment of spouses, and allowing for no-fault divorce, divorce rates sky-rocketed [60,83]. Research suggests negative effects on average of divorce on the health and well-being of spouses [15,20,70,[76], [89]] and perhaps even more notable negative effects on children [[10], [11], [12],^18^,^24^,^34^,^87^]. This does not mean that divorce is never the right answer; there are faults of unfaithfulness or abuse, for example, which may render divorce the best option; and single parents and their children should be strongly supported, like all others, and can be enabled to flourish, especially with such strong support. However, an altered understanding of marriage, focused more exclusively on the bond between spouses, makes it far more common for divorce to seem like the right way to proceed. The redefinition thereby effectively alters opposite-sex spouses' understanding of marriage as well. A person's willingness to stay in marriage may become more focused upon personal fulfillment, and less focused upon children. Moreover, with a conception of marriage more detached from children, it may become difficult for some to even understand the point of getting married, and the rate of opposite-sex marriage may itself then decline, and the openness to children outside of the context of marriage may increase [68]. It is not clear, if marriage is just about love between partners, why it is important to regulate marriage at all [31]. As a complex illustration of several of these points, and their interplay, some time ago, a colleague of mine was engaged to her partner, who was the father of their two children; during the course of their engagement, he told her that he was no longer in love and was leaving. If marriage is principally about the fulfillment of spouses, his response is understandable; his response was not necessitated by such an understanding, but it coheres with it.

Finally, the effective removal of reproduction and care for children from being embedded within the definition of marriage itself I think also shifts societal focus away from children's welfare and towards adult fulfillment. This shift arguably affects a host of issues ranging from teacher pay, to tax policies, parental leave and childcare policies, policies concerning children's use of social media, and decisions concerning various COVID response options and how these affect different age groups ([[32], [49], [50], [56], [59], [73]]). These shifts are not inevitable, and can be resisted, and there are undoubtedly numerous other societal forces at play in shaping these policies, but I believe an altered definition of marriage focused more exclusively on spouses naturally shifts societal focus away from children, and the priority given to the welfare of children. The question is not so much how marriage can or might function in any given relationship (same-sex couples can certainly lovingly care for children), but rather how marriage is functioning as an institution.

I do not see how these three points above could be completely irrelevant to childhood well-being, but it is nevertheless still conceivable that their corresponding effect sizes might be negligible. Some of the views above are thus at least partially open to empirical refutation. To gain insight as to how these three considerations above may relate to childhood well-being, one might attempt to collect data over time oriented towards questions like: What proportion of children are living in marriages with both biological parents? To what extent are spouses committed to remaining in a marriage for the sake of children? To what extent is society giving priority to the well-being of children within its policies? Data collection may give insight into how these matters are changing over time. However, as I have commented elsewhere, traditional cohort-based methods for causal inference are not particularly well-suited to provide evidence for the effects of cultural movements, and other methods of cultural or historical analysis may be more appropriate [75].

I do not believe that the question of same-sex marriage is completely responsible for the various cultural changes indicated above, nor do I think that these cultural changes were intended by many of those advocating for same-sex marriage. Moreover, as noted above, I do think each of the two alterations to the definition of marriage were in principle independent. However, I also think that each independently weakens the link of marriage with reproduction and the care of children. In many ways, same-sex marriage was simply the conclusion of alterations, that began with no-fault divorce, towards a definition more exclusively focused on the fulfillment of spouses. Nevertheless, I still do not see how the traditional definition of marriage can be modified without, in one way or another, re-orienting it away from children. And I do believe that this re-orientation has consequences for children's well-being.

The *amicus* brief did, however, also acknowledge that a revised definition would provide various practical and social benefits to those in same-sex relationships, that could be weighed against the alleged claims concerning the welfare of children, and that there was room for genuine disagreement. The brief further acknowledged the long and tragic history of cruelty towards same-sex attracted persons; and I certainly think such cruelty is wrong. The brief further discussed the need to affirm equal dignity of all people, and the possibilities of ensuring civil rights, and a respect of the importance of the relationships of same-sex attracted people, regardless of the ultimate societal definition of marriage. However, given the various trade-offs at play, it was again argued that the weighing of these considerations ought to be left to the states, so as to adjudicate between the various matters under discussion. I do not expect readers to agree with my positions, but I hope that the exposition above at least helps with understanding how I can hold my position without having an animosity towards same-sex attracted people. I believe LGBTQ+ people should be treated civilly, and should be supported and cared for, as should all people; their human rights and personhood should be respected.

I will now, far more briefly, consider the positions put forward in the *Psychology Today* blog post [79] and the *JAMA Psychiatry* commentary [2]. The *Psychology Today* blog post raised the question of whether introducing matters of gender identity in kindergarten was conducive to well-being. When my wife and I were touring kindergarten and pre-kindergarten classrooms in the Cambridge public schools, we were surprised to observe a teacher reading the book, “Who Are You? A Kid's Guide to Gender Identity,” to pre-kindergarten students at circle time, suggesting to the students that their parents may have wrongly guessed the gender of their child, and providing a gender wheel in the back of the book to help the students explore a number of different options. Personal distress over gender identity is a real and difficult issue, and I do not pretend to know the right care approaches or interventions for those suffering from this at various life stages. I do, however, have concerns about the age at which these matters are being addressed in the general curriculum. It is not clear to me that including this book in the pre-kindergarten curriculum, at the age of 4, is more conducive to societal well-being than dealing with questions of gender identity on an individual, or classroom, as-needed basis, if or when required. I have concerns about whether pre-kindergarten curricula on gender identity might be creating gender dysphoria, rather than alleviating it, and whether similar phenomena might be at play in other contexts as well [47]. It does not seem unreasonable to raise these concerns, and that is what the blog post did. I think that there needs to be more open discussion in academia, and in society, about these matters. Most people, even those who are deeply concerned, seem very uneasy discussing these issues, for fear of being attacked for simply raising them. Colleagues at Harvard, ranging from an expert in child development to a clinician providing mental health care for teenage girls, have told me that they are uncomfortable sharing their concerns on these matters in many or most settings at Harvard. An evolutionary biologist at Harvard likewise recently came under attack because she explicitly stated that sex was biological and binary [3], even though she also noted that we can nevertheless respect a person's gender identity. The attack was sufficiently severe, and the administration's response sufficiently weak, that she eventually felt she had no choice but to resign. Rather than open discussion, it seems we are often now relying on anonymous articles [26], or brave, and subsequently vilified, authors [69,72] and whistle-blowers [66] to raise alternative viewpoints. One may strongly disagree with their positions, but it is not unreasonable to raise the questions. I think that there are real and reasonable concerns about the welfare of children embedded in these questions.

In the *JAMA Psychiatry* commentary [2], I argued that the abortion and mental health literature had been weaponized by both sides of the abortion policy debate; that the moral contours of the policy debate lay elsewhere; and that the abortion and mental health literature should thus be more oriented towards providing for the mental health needs of women regardless of their views. I had pro-choice colleagues write to me indicating, “I find nothing even to disagree with” or to say that the commentary was “thoughtful, beautifully written, and very well balanced.” The commentary was sufficiently centrist that the *Harvard Gazette* solicited and ran an interview article with me on it [65]. It may well still come, but no one at HSPH, or within the rest of Harvard, has to date noted to me anything in the commentary with which they disagree. I had correspondence with an epidemiologist at a different institution who took issue with the sentence, “The one meta-analysis on abortion and depression has come under reasonable critique; yet critics have not produced an alternate meta-analysis and the 10-year-old study may still be the best quantitative-synthesis estimate available.” To the best of my knowledge, this is still the only meta-analysis, but the argument in the commentary in no way hinges upon this point. Nevertheless, in spite of trying to find common ground in the commentary, and, with some colleagues at least, evidently succeeding at this, the article was regularly referenced in the Twitter posts; and it was among the writings that were deemed as problematic by some of the HSPH students, faculty, and administrators. As best as I can tell, the reasoning was that because I did not explicitly affirm a pro-choice position or because one can read between the lines to infer my position on abortion, that this, rather than my actual words in the commentary, was problematic. It is true that I believe that abortion typically involves someone acting, as an individual, to end human life as the intended result, and thus constitutes action that is wrong, and a violation of human rights – the right to life of the fetus [44]. This not infrequently occurs because women's financial, relational, and emotional needs are not met, and so I believe there is also a societal culpability for this as well. With regard to policy, while I certainly do not think the laws are irrelevant, I believe more work should be oriented towards creating a positive culture of life that welcomes and sees the value of all children and all life, and also towards structuring our societal life so as to better provide for the economic, emotional, and social needs of women so that pregnancies are less often unwanted [28,81,86].

My hope in this section was to explicate my positions in slightly greater detail and give some of the reasons for those positions. I do not expect that the explications above will necessarily persuade, but I hope they will at least help colleagues understand why I hold the positions I do. Ultimately, I hold each of these positions on account of the welfare of children: what I see as unborn children, children in schools, and children within families. These are of course not the only issues that threaten child welfare. The Catholic Church is itself sadly culpable for a long history of abuse. I strongly believe that addressing this too should also be a public health concern [39,78], and that while considerable progress has been made in prevention [30], greater accountability for those who perpetrated and covered up these incidents is still needed. In any case, likely due to a myriad of causes, the data indicate that the well-being of young people has been in notable decline [19,23,58]. Children are among the most vulnerable in society and I do not think their well-being has received adequate attention in public health. During the twenty years since I began as a graduate student at HSPH, the Department of Maternal and Child Health was dissolved (and its faculty mostly subsumed into what is now the Department of Social and Behavioral Science) and during this time the Sexual Orientation and Gender Identity and Expression Health Equity Research Collaborative was established. Research on both of these broad topics should arguably be well represented at a school of public health. However, the decreasing prominence of one, along with the increasing prominence of the other, I think is indicative of the shifting priorities in academic public health. I believe we have been neglecting the well-being of children and young people. The potential causes of this are diverse, but I believe that each of the three issues described above is a part of the story.
